# Human–Machine Collaborative Learning for Streaming Data-Driven Scenarios

**DOI:** 10.3390/s25216505

**Published:** 2025-10-22

**Authors:** Fan Yang, Xiaojuan Zhang, Zhiwen Yu

**Affiliations:** 1 School of Computer Science, Qinghai Normal University, Xining 810016, China; zhxj@qhnu.edu.cn; 2 The State Key Laboratory of Tibetan Intelligent, Xining 810016, China; 3 School of Computer Science, Northwestern Polytechnical University, Xi’an 710129, China; zhiwenyu@nwpu.edu.cn

**Keywords:** human–machine collaborative learning, deep learning, streaming data-driven, human intelligence

## Abstract

Deep learning has been broadly applied in many fields and has greatly improved efficiency compared to traditional approaches. However, it cannot resolve issues well when there are a lack of training samples, or in some varying cases, it cannot give a clear output. Human beings and machines that work in a collaborative and equal mode to address complicated streaming data-driven tasks can achieve higher accuracy and clearer explanations. A novel framework is proposed which integrates human intelligence and machine intelligent computing, taking advantage of both strengths to work out complex tasks. Human beings are responsible for the highly decisive aspects of the task and provide empirical feedback to the model, whereas the machines undertake the repetitive computing aspects of the task. The framework will be executed in a flexible way through interactive human–machine cooperation mode, while it will be more robust for some hard samples recognition. We tested the framework using video anomaly detection, person re-identification, and sound event detection application scenarios, and we found that the human–machine collaborative learning mechanism obtained much better accuracy. After fusing human knowledge with deep learning processing, the final decision making is confirmed. In addition, we conducted abundant experiments to verify the effectiveness of the framework and obtained the competitive performance at the cost of a small amount of human intervention. The approach is a new form of machine learning, especially in dynamic and untrustworthy conditions.

## 1. Introduction

Deep learning has been remarkably successful in many fields, and the process has shown superiority in various real-world applications over most machine approaches. Although the accuracy of the executed task is gradually improved by the neural network architecture, the reason for the output is still not completely clear and understandable. Human–machine fusion intelligence is a kind of novel intelligent computing method which integrates human intelligence and machine intelligence in an effective way to deal with complex tasks. In contrast, when either human or machine intelligence performs work individually, neither achieved ideal performance. Machine learning and deep learning show great power in many fields, such as object detection, face recognition, natural language processing (NLP), and automatic speech recognition (ASR). As we enter an era of growing data rates and volumes, it may produce several terabytes of data each day of a public surveillance camera or thousands of terabytes produced by an ecology project annually. Zooniverse is adopting an increasingly sophisticated system design that leverages the complementary strengths of humans and machines. This integrated approach is engineered to maximize both operational efficiency and human engagement [1]. Human intelligence and machine intelligence are complementary due to their natural characteristics; it is a novel pattern to address specific complicated tasks. In this mode, humans can perform subtasks of logical reasoning, whereas machine can process repetitive and tedious calculation tasks. The theory of human–machine hybrid intelligence is in its infancy and may become a common way to resolve issues in the future.

Some researchers have focused on the work of human–machine cooperation in recent years, which leverages the advantage of human feedback and machine computing power to make the final decision. Zhou et al. [2] introduced a framework that integrates uncertainty and performance metrics into AI-advised decision making to enhance user trust. Carrie et al. [3] provided an approach to retrieve visually similar medical images from previous patients to obtain a more reliable medical decision with a new patient. Dominik et al. [4] propose hybrid intelligence that takes advantage of artificial intelligence and human intelligence to solve tasks such as object detection and speech recognition. When the aim is to find a suspect from several hours of video recordings, it is labor-intensive for persons gazing at the monitor, lasting for four to five hours or much longer, and it is prone to make mistakes. In another mode, collecting and labeling a great amount of samples, and then training a model to detect the query person, the model is conducted on the video sequences in order to obtain each frame of the suspect. There is some error detection inevitably due to different illumination or the suspect being shielded by other objects (trees, crowds, and cap on the head). The combination of human intelligence and machine intelligence is a significant way to address complicated tasks. Humans are good at studying from a few instances and giving their judgment; especially in dynamic environments, people have a natural ability to deal with uncertainty. Machines are capable of executing tasks in a given program; however, machines cannot provide a clear decision when facing variations due to the lack of appropriate measures. In this paper, we assume that humans and machines are peer computing units in a task which would be processed.

Human–machine collaborative learning is a novel attempt to solve complex tasks, especially for streaming data-driven scenarios such as, for instance, video anomaly detection, person re-identification, and domestic sound event detection. In this paper, we explore the common characteristics of the theory on the fusion of human intelligence and machine intelligence, but it poses difficult challenges:

- Fusion of human intelligence and machine intelligence: Human beings make decisions based on their knowledge and experience; in contrast, machines provide automatic output through coded programs. There is a natural difference between how humans and machines address issues; thus, it is important to explore how we can efficiently integrate these two different kinds of computing units together to perform complicated tasks.
- Model construction of humans and machines: Due to their different computing patterns and conditions, how can humans and machines cooperatively work in a common space when giving a task, analyzing the requirement of a task, and adopting a proper strategy to work? The aim is to build a model that contains humans, machines, a collaborative mode, and all the related algorithms.
- Design rules for human–machine collaborative learning: Although humans and machines work in a common space, when it comes to which computing units should be executed first in a phase, there should be a basic strategy to plan the first step. The goal is for the critical rules to ensure that the whole system runs smoothly.
- Build evaluation criteria: To the best of our knowledge, there is a lack of performance evaluation for human–machine cooperative work. One of the reasons is that human performance is hard to clearly assess because human work efficiency is susceptible to the individual’s mental state at a given point in time; however, the evaluation of machine computing performance is far more straightforward. Therefore, it is important to consider special discrimination and provide efficient indicators for the human–machine collaborative learning system.

To address the challenges mentioned above, we proposed a new collaborative human–machine learning framework for streaming data-driven scenarios (HMLSDS), which can cooperatively work to deal with tasks by humans and machines in an efficient pattern. Our framework has three parts: the initialization part, the computing part, and the evaluation part. We verified our framework in the context of different scenarios, including video anomaly detection, person re-identification, and sound event detection. The experimental results demonstrated that our framework can achieve fairly satisfactory performance in streaming data-driven scenarios.

The main contributions are summarized as follows:

- To the best of our knowledge, this is an exploitative work on human–machine collaborative learning for data-driven scenarios. In addition, we propose a complete cycle from task processing to performance evaluation.
- We provide a theoretical analysis for human–machine collaborative learning, which represents a paradigm shift from fully automated systems to synergistic partnerships. Within a subtask, human computation can influence the machine’s outcomes via flexible modes of interaction.
- We designed an evaluation criterion for human–machine collaborative learning through the amount of human workload and improvement percentage time to show a correlation between humans and the task performance.

## 2. Related Work

Hybrid augmented intelligence is one of the most important directions in artificial intelligence [5]. Many countries have studied the fusion of human and machine intelligence since 2016. There are three types of research related to human–machine collaborative learning, which integrates human intelligence into machine computing: (1) human in-the-loop machine learning, (2) interactive machine learning, and (3) human learning.

### 2.1. Human-in-the-Loop Machine Learning

Human-in-the-loop machine learning (HITL) is a smart model of human–machine interaction. In this model, humans are part of the system; people use their expertise and experience to provide further confirmation or feedback. More and more researchers have proposed approaches to tackle the challenge of fewer training samples that incorporate human knowledge into modeling. An approach named “human-in-the-loop” is used to address complex tasks, such as person re-identification [6], medical diagnosis [7], and anomaly detection [8]. Although machine learning and deep learning have been applied in many fields and achieved remarkable performance, there are some shortcomings, such as the explanation of the outputs, the large amount of labeled training samples and hundreds of millions of neural network parameters. For a special task, there would be no adequate samples to train a competitive model. The progression of HITL ML has three objectives: (1) obtain a higher model accuracy; (2) bring the model up to an acceptable precision as quickly as possible; and (3) incorporate human intelligence and machine intelligence into a common space for an optimized computing combination. There have been several successful models, such as Transformers [9], Bidirectional Encoder Representations from Transformers (BERTs) [10] and Generative Pre-trained Transformer (GPT) [11]. Many researchers have suggested using fewer data to obtain better performance. Self et al. [12] proposed an interactive parameter adjustment mode, allowing users to directly participate in the process. In addition, they emphasized the value of such human–computer interaction for user-centric analysis. Zhang et al. [13] proposed an HITL method for data annotation and human selection of the optimum sample in an iterative labeling mode for entity extraction. Cheng et al. [14] proposed a hybrid crowd–machine learning platform to predict a person whether is lying; it guides crowds to nominate effective features and then uses machine learning techniques to aggregate marked labels for more accurate prediction. Researchers have found that incorporating human knowledge and experience into teaching machines improves results, and reaching a higher level of processing performance.

Contemporary research in human-in-the-loop computer vision explores two key areas: the use of weak labeling for model feedback and the design of unified and intuitive interfaces for user intervention [15]. Currently, researchers in computer vision are mainly exploring how to use weak labeling to provide human feedback. In addition, they offer a consistent and intuitive interaction model for user guidance. Liu et al. [16] proposed a human-in-the-loop model based on reinforcement learning, which overcomes the limitation of pre-labeling and updates the model through continuous data collection. Butler et al. [17] proposed a micro-expression recognition approach based on the HITL system, which provided a smart interface for the manual operation of automatically processed labels, guaranteeing the accuracy and usability of the extended dataset. In addition to data preprocessing and data annotation, iterative labeling is a vital operation of model construction. It has been discovered that the quality of the data has a direct impact on the final execution performance. For a new task, if you hope to achieve good results, a large quantity of labeled data would be needed. The key point of HITL-based methods is to find critical samples and proper human intervention. Yu et al. [18] provided an automated labeling scheme, releasing human workload, using the human-in-the-loop mode.

### 2.2. Interactive Machine Learning

Interactive machine learning (IML) is the design and implementation of algorithms and intelligent user interface frameworks that facilitate machine learning with the help of human interaction. IML leverages human feedback during training to improve model performance [19], which has gradually become a hot research point [20,21]. For example, using user-given cases, Amershi et al. [20] presented an algorithmic approach to analyze new friend groups on social networks, while Fogarty et al. [21] explored an interactive paradigm in which users directly teach new concepts to a search engine. Amershi illustrated the impact of humans on IML and testified that they can perform tasks more efficiently [22]. Sacha et al. [23] pioneered a conceptual framework for visual analytics that identifies critical scenes to seamlessly merge ML with human-informed cooperative visualization. Justin et al. [14] contributed to the “Flock” classifier, which accelerates model generation in crowd–machine systems and thereby optimizes computational execution and augments human judgment effectively. To integrate human knowledge into machine computation, Doris et al. [24] provided a theoretical model to accelerate human-in-the-loop machine learning. Holzinger [25] formally defined IML as algorithms that interact with agents (including humans) to optimize their learning processes. This paradigm leverages human experience to solve challenging problems, such as subspace clustering and protein folding, by heuristically reducing exponential search spaces. Based on this, the literature [26] provided experimental insights by integrating human intelligence into an Ant Colony Optimization (ACO) framework, establishing an effective “multi-agent in the loop” system. Complementing these approaches, Berg et al. [27] introduced an intuitive interactive tool that enables domain experts, such as biologists, to use ML-based image analysis without deep computational knowledge. Teso et al. [28] designed a framework of interactive interpretative learning at each step, in which learners explain their queries to users, and users interact by answering queries and correcting explanations. They use text and image categorization experiments as user studies that demonstrate improved predictive and interpretive abilities as well as trust in learning models.

### 2.3. Human Learning

Human cognition is distinguished by an innate versatility for learning and adaptation that appears to be unique in the animal kingdom. Although the acquisition of specific knowledge and skills is profoundly influenced by the environment, the human capacity to adapt to a wide range of contexts far exceeds that of other species [29]. The formal scientific inquiry into human learning originated in the late 19th century. Ebbinghaus [30] postulated the learning curve based on empirical data showing a leveling in learning rates over time. Subsequently, Kotovsky and Simon [31] conducted foundational large-scale analyses of learning patterns. Modern understanding is guided by three central theoretical frameworks: cognitive psychology, social cognitive theory, and sociocultural theory [32]. The mechanisms of implicit learning were further examined by Sharks et al. [33], who synthesized evidence from domains such as subliminal learning and conditioning.

Thus, learning is a multifaceted process that involves not just the mastery of skills and information but also the assimilation of values, attitudes, and emotional patterns.

This study investigates human–machine collaborative learning under small-sample constraints, benchmarking it against supervised machine learning. The work is more closely aligned with the field of cognitive psychology. Each person has a different work performance on the same task due to their different backgrounds of expertise and experience. In addition, the same person doing a job in a different mental state would obtain a distinct result. Theoretical frameworks from this field inform our understanding of human learning patterns. Cognitive psychology is “the study of how people perceive, learn, remember and think about information” [34]. The study of cognitive psychology involves the study of psychological phenomena such as visual perception, object recognition, attention, memory, knowledge, speech perception, judgment, and reasoning. To explain this phenomenon, cognitive psychology turns to neuroscience and its knowledge of brain function. Illeris [35] aimed to establish the basis for unified learning. His work delineates the core components and knowledge domains required to build a theory that is comprehensive and coherent, accounting for the full spectrum of psychological, biological, and social conditions involved in learning. Employing three different experimental paradigms, Kuhl et al. [36] conducted a pioneering comparison of learning performance between humans and supervised machine models when training data are scarce. Humans seem to learn more from a small number of examples than machines. Studying the mechanism of the human is a significant research area in the development of modern artificial intelligence.

## 3. HMLSDS Framework

In this section, we elaborate on our human–machine collaborative learning framework for streaming data-driven scenarios. In terms of concreteness, we describe the special characteristics of a human and machine to perform a specific task in Section 3.1. Then, we present the HMLSDS framework in Section 3.2. The critical rules for HMLSDS are designed in Section 3.3.

### 3.1. The Characteristics of Human and Machine

In intelligent systems, specialized models are often designed to address specific issues effectively. However, when faced with a new task, these models tend to fail due to differences in settings and requirements. It would be beneficial to establish a general framework that enables a model to handle data-driven tasks by leveraging human expertise and experience without the need to reconstruct a new system from scratch. Such a framework would allow humans to respond to dynamic situations, integrating human judgment with machine computation to create a complementary effect throughout the task-executing process. Consider a common scenario often depicted in television or film: investigating a homicide or identifying a suspect. Manually searching through vast amounts of surveillance footage to accurately pinpoint a suspect is highly labor-intensive. Although several classical deep learning methods have achieved competitive detection performance, they still suffer from false alarms and missed identifications. These errors arise due to factors such as varying lighting conditions, obstructions, and changes in the appearance of the same person across different outfits.

Human beings and machines utilize distinctive characteristics to engage with the same work. Humans have creativity, empathy, reasoning, analysis flexibility, and common sense; these features are unique for each person. However, fast speed, large storage, pattern recognition, and consistency are vital character properties for intelligent machines. In recent years, more and more countries have begun to study human–machine collaborative intelligence.

**Figure 1:**
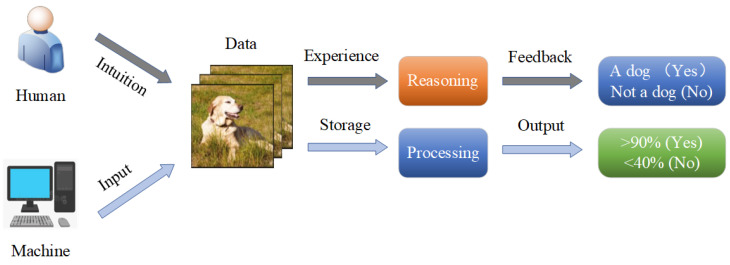
Human and machine computing process.

Through observation and deep thinking, we designed a human and machine computing process, which is illustrated in Figure 1. Humans and machines work in a common workspace; when dealing with the same task, humans and machines compute in different ways, respectively. For human beings, they observe a task intuitively, using their experience to compute or reason, and giving the final feedback. For the machine, it first needs the input of the primary data, and then it makes use of processors and storage memory for further processing. Finally, it outputs the result in probability format.

It is an iterative and mutual learning method that integrates the cognitive reasoning of the human and the intelligent computing of the machine, in which the human and machine execute according to the rules, interact and cooperate with each other to give full play to their respective advantages for a specific streaming data-driven task. For an unsolved data-driven task, humans and machines work in a cooperative mode: machines provide powerful computing ability to process the most repetitive steps, whereas humans leverage their expertise and experience to guide the machines for a better direction of optimization or give their judgment for a complicated situation that the machine cannot address properly. A streaming data-driven task means that the final outcome is computed by the original data and then executed in a human–machine cooperative mode. In the whole performance cycle, the data are vital; in other words, the data make each processing stage run forward; without the data, it would do nothing. In contrast to this category, another type of task depends on specific scenarios, such as auto-driving and virtual reality applications.

There are three stages in which collaborative learning can be performed between humans and machines: (1) collecting samples means selecting representative to reduce the number of training sets, (2) incorporating human intelligence to adjust the model and iterate in a closed-loop form; and (3) analyzing the decision-making output by estimating whether the machine algorithm’s output is correct or contains some wrong results and then giving a highly reliable decision.

### 3.2. HMLSDS Framework

**Figure 2:**
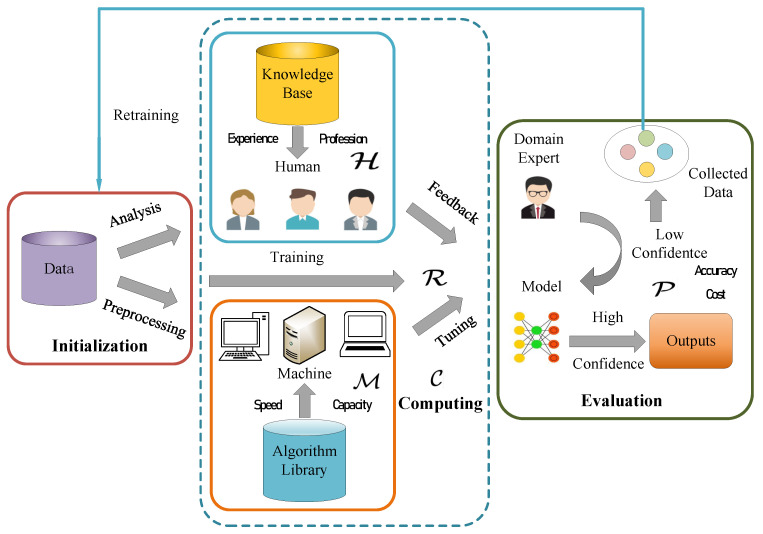
Human–machine collaborative learning framework for streaming data-driven scenarios.

It is important to fuse human cognitive reasoning and intelligent machine computing for subtasks and collaborating in a complementary way. Our proposed framework is illustrated in Figure 2. The framework contains three parts. (1) The first part is initialization, in which data are prepared to be computed for a certain task. At this stage, both humans and machines would perform a few tasks (human analysis and/or machine preprocessing, such as data cleaning, basic model training, and special sample collection). (2) The second part is the computing part; here, the machine selects a specific algorithm from the algorithm library, whereas the human has a knowledge base. The model is run in an iterative way until a predefined threshold is met or budgets are exhausted. (3) The third part is an evaluation part that evaluates the efficiency of human–machine learning. Furthermore, the most important thing is that a proportion of the low-confidence data will be collected for further training in an iterative pattern. The collected samples are mainly hard ones, such as are heavily shielded crowds or someone that is not clear, but in the same dressing style similar to the target.

Compared with the usual ML procedure, our framework has a distinctive feature which fuses human intelligence and machine intelligence together; the advantage is that we use less human workload to obtain higher model accuracy. Human can clean data in the initialization stage; at the same time, domain experts can give their feedback to the low-confidence output of the model and collect these data samples into the training phase to fine-tune the model. Through extensive experiments, our framework increases in precision with a human workload of no more than 20%. At the same time, human–machine learning can find the complex situation and take measures to prevent the model from going on a terrible performance trend; this is more flexible and efficient.

Our framework is implemented in a five-tuple $L=\left\{H,M,R,C,P\right\}$, where H represents the human computing unit, M represents the machine computing unit, R represents the computing relationship between the relevant human being and the machine computing unit, C represents the mode of collaboration between humans and machines, and P represents the performance of human–machine learning execution. For a common computing unit U which can express that a human or a machine has a set of capabilities, $Cp^{U}=\left\{\left(cp_{1},x_{1}\right),\left(cp_{2},x_{2}\right),\ldots \right\}$. The capability type $cp_{i}$ defines the kind of function that the computing units have or are endowed with, and $x_{i}$ represents its capability level. There are two types of capability level, a Boolean capability (i.e., it has or does not have specific capability) and a continuous capability level, for example (0, 1], which defines a floating value representing the quality of the computing unit. These values can be calculated on the basis of a qualification test or on a statistical measurement such as Amazon Mechanical Turk.

We designed the detailed information of the framework as follows:

H stands for human computing unit; it can be only one expert $h_{i}$ who is familiar with a specific field and has extensive experience, or it would be a group of workers or experts gh. For each human, different abilities belong to different people.

M stands for the machine computing unit; as mentioned above, it is similar to the human computing unit in that each machine $m_{j}$ has its own performance parameters.

R stands for the computing relationship between the relevant human and the machine computing unit. If a human computing unit $h_{i}$ and a machine computing unit $m_{j}$ work in a parallel mode, the relationship uses “0” to represent this mode. When an intelligent machine performs the task, human experts can observe the intermediate result. Another relationship is “1” to represent the serial mode; it is more common when the machine completes the computing task and the human takes measures on low-confidence output. We formulate the calculation relationship $R_{i}$ as follows.

(1) $$R_{i}=\{\begin{array}{c} 0,\;Parallel.\\ 1,\;Serial. \end{array}$$

C stands for the collaboration mode of human and machine computing units. There are three types of cooperation. We use $C_{k}$ to represent the different modes. “1” represents a human computing unit that interacts with a machine computing unit. In this mode, an interactive interface is designed, in which human experts can observe the intermediate output and perform some operations, such as “mark”, “zoom in”, and “zoom out”. “2” represents humans giving feedback on the machine computation output; humans can then learn new knowledge from the machine and then give some advice to promote model construction. For example, in person re-identification tasks, humans find more overlap in some crowded frames and set the IoU to a smaller number, further reducing occlusions. “3” represents an augmented mode in which humans and machines can work collaboratively to enhance performance improvement; especially, human experience can augment the new method to address the current complex situation that only the machine can solve. For instance, in the sound event detection task, several sounds are mixed in a short slot; due to the different frequencies of each sound, the model can be designed to use several frequencies to discriminate coarse-grained classes.

(2) $$C_{k}=\{\begin{array}{c} 1,\;Interactive\;mode.\\ 2,\;Feedback\;mode.\\ 3,\;Augmented\;mode. \end{array}$$

P stands for the performance of the human–machine learning execution. We consider three vital performance metrics: the accuracy of the final task is Acc, the human involvement of a given task is expressed as Workload, and a special metric is Cost, which stands for a ratio of human workload to task accuracy—in other words, when increasing the amount of human involvement prompts the final precision improvement.

To avoid ambiguity, we explicitly defined the two-core metrics of the cost/benefit analysis, aligning them with standard practices in streaming ML research:

Human effort: quantified as the total labeling time (in person-hours) required to prepare training data for each baseline method and our proposed method. For consistency, we used a team of five annotators (with 2+ years of experience in video/image/audio labeling) and measured the time per sample;

Accuracy: task-specific metrics to reflect real-world utility:

(1) Video anomaly detection: F1-score (area under the curve, AUC) for the location of anomaly events.

(2) Person re-identification: mean Average Precision (mAP) across five camera views.

(3) Sound event detection: event-based F1-score (counting for both category accuracy and temporal alignment of detected events).

For a specified task, we can represent the execution performance $P_{k}$ as follows.

(3) $$P_{k}=\{\begin{array}{c} Acc,\;Final\;task\;accuracy.\\ Workload,\;Human\;involvement.\\ Ra,\;Ratio\;of\;the\;Workload\;to\;the\;Acc. \end{array}$$

In the initialization part, the basic task requirements would be known, such as task type (classification task, identification task, generation tasks, etc.), desired accuracy, and expected computation time. After obtaining the essential information, the human can analyze the complexity of the computation of the task. In most cases, the collected datasets conclude samples that are more or less noisy, and humans and machines can discard or refine them. Then, the preprocessed data can be computed in the subsequent steps.

In the computing part, which is a vital section of our framework, machines run a specified algorithm to address the preprocessed data utilizing a server with a GPU or a common PC. Humans with sufficient knowledge or experience can observe the intermediate output and can provide feedback to promote the rate of model convergence. For example, in the identification task, humans can choose incorrect classification samples as useful samples back to the training stage to train a robust model for testing.

In the evaluation part, an expected threshold would be set (according to the accuracy of the advanced algorithm which has been published). When the accuracy is larger than the threshold, it would output directly. For low-confidence output, the domain expert would give a confirmation or correction to enhance the final precision. We focus on select sample numbers and the ratio of the amount of human workload to the task performance as the main evaluation index.

Our proposed framework contains three parts as mentioned above; it is not isolated but runs iteratively in a loop. In the training phase, some special samples can be obtained for better model construction based on expert feedback. In the testing phase, it is not a one-time process just to give the final output; instead, a proportion of the low-confidence output data should be collected as meaningful samples input for fine-tuning training. Human and machine computing units work cooperatively in a common space in a specific collaborative style.

### 3.3. Rules for HMLSDS

**Figure 3:**
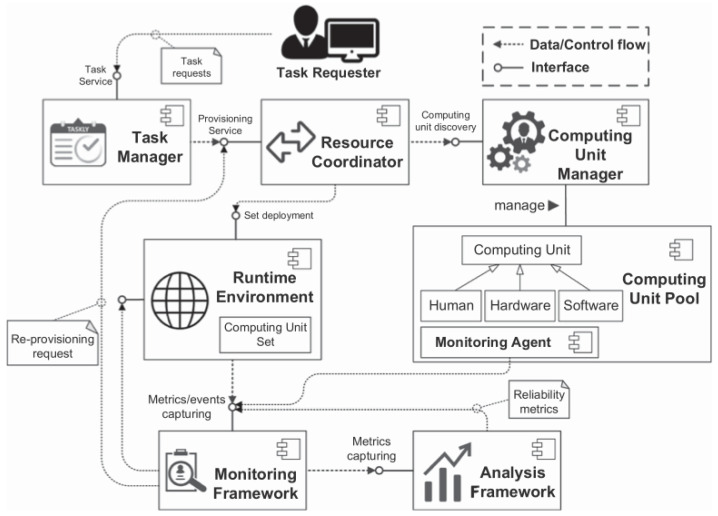
Workflow of the framework runtime.

In this section, we design basic rules for the framework to ensure that it can work smoothly as often as possible, mainly referring to the task allocation mechanism and conflict resolution to address human and machine judgements. Furthermore, model adjusting based on human feedback is a vital issue to be addressed. The workflow of the framework runtime is shown in Figure 3. The runtime starts when a new task arrives and the task requester sends messages to the task manager, which is responsible for constructing a queue to be processed. The prepared request is then delivered to the resource coordinator, who is responsible for assembling a set of computing units. Potential computing units are selected by a computing unit manager. All of the computing units are virtualized in a computing unit pool to be chosen by the computing unit manager. Similarly to the computing unit manager, runtime environments are deployed to certain tasks. The monitoring framework through an interactive interface provides the current state of the process. Finally, the analysis framework displays critical performance metrics. With a clear operational process in place, the task can be executed effectively. Depending on the application (and the research groups), different abstractions and solutions will be used [37].

Mathematical modeling of framework

We formalized the framework’s solution with a clear mathematical notation as follows:

1. As the core mathematical formulation of our HMLSDS framework, the dynamic feature alignment module with weakly supervised learning is designed to address the “non-stationary” nature of streaming data (i.e., concept drift, where data distribution shifts over time). We formalized this problem and the framework’s solution with a clear mathematical notation as follows:

(1) Problem definition: Modeling streaming data distribution shifts, we define the streaming data at time step t as a sequence $D_{t}={\left\{\left(x_{t,i},y_{t,i}\right)\right\}}_{i=1}^{N_{t}}$, where $x_{t,i}\in R^{D}$ is the i-th input sample (e.g., video frame feature, audio spectrogram feature) at time t with dimension D; $y_{t,i}\in Y$: the weakly supervised label of $x_{t,i}$ (e.g., “anomaly present” for video, “target person present” for person re-identification)—distinct from full supervised labels (which require fine-grained localization/timestamps); $N_{t}$: the number of samples arriving at time t (varies for streaming data, e.g., 30 samples/second for 30 fps video). The core challenge of concept drift is modeled as a time-varying probability distribution: $P_{t}\left(x,y\right)\neq P_{t-\tau }\left(x,y\right)\;\mathrm{for}\;\tau >0$ where $P_{t}\left(x,y\right)$ is the joint distribution of input x and label y at time t. Traditional static models (e.g., supervised ViT-B) assume $P_{t}=P_{t-\tau }$, leading to performance degradation when drift occurs.

(2) Framework objective function: Balancing adaptation and stability to address drift while avoiding "catastrophic forgetting" (losing knowledge of past data), our framework optimizes a hybrid objective function that combines the following: current-time supervision loss (to adapt to the latest data distribution); past-feature alignment loss (to retain consistency with historical features); and weak-label regularization (to leverage sparse weak labels efficiently).

The full objective at time t is the following:

(4) $$L_{t}=\alpha \cdot L_{\sup}\left(f\left(x_{t,i};\theta_{t}\right),y_{t,i}\right)+\beta \cdot L_{\mathrm{align}}\left(\theta_{t},\theta_{t-1},F_{t-1}\right)+\gamma \cdot L_{\mathrm{reg}}\left(\theta_{t}\right)$$

where $\theta_{t}$: model parameters at time t; $f(\cdot ;\theta_{t})$: model prediction function (e.g., feature extractor + task-specific classifier); α, β, γ: hyperparameters (set via cross-validation to 0.6, 0.3, 0.1, respectively) that balance the three loss terms. The definitions of each loss component are provided in the following with the derivations linking them to drift mitigation.

2. We created a detailed derivation of key loss components. To avoid “black-box” modeling, we derived each loss term with theoretical justification for its role in streaming tasks:

(1) Supervision loss ($L_{\sup}$): Adapting to current data for weakly supervised labels (e.g., binary “event present/absent”), we use a weighted cross-entropy loss to account for label uncertainty (weak labels are less precise than full labels). For a sample $x_{t,i}$ with a weak label $y_{t,i}\in \left\{0,1\right\}$,

(5) $$L_{\sup}=-\frac{1}{N_{t}}\sum_{i=1}^{N_{t}}\left[w_{t,i}\cdot y_{t,i}\log\left(f\left(x_{t,i};\theta_{t}\right)\right)+\left(1-w_{t,i}\right)\cdot \left(1-y_{t,i}\right)\log\left(1-f\left(x_{t,i};\theta_{t}\right)\right)\right]$$

where $w_{t,i}\in \left[0.8,1.0\right]$ is a confidence weight for the weak label—higher weights are assigned to labels verified by 2+ annotators (reducing noise from weak ambiguous labels). Theoretical rationale: This loss ensures that the model learns the latest data distribution $P_{t}\left(x,y\right)$, addressing “sudden drift” (e.g., a new type of anomaly in video streams).

(2) Loss of feature alignment ($L_{\mathrm{align}}$): To mitigate catastrophic forgetting to retain knowledge of past data, we align the feature space of the current model with the feature space of the previous time step (t−1). We define $F_{t-1}={\left\{f\left(x_{t-1,i};\theta_{t-1}\right)\right\}}_{i=1}^{N_{t-1}}$ (historical feature set) and compute the Mahalanobis distance between the current and historical feature distributions (to measure the severity of the drift).

The alignment loss is shown below:

(6) $$L_{\mathrm{align}}=\frac{1}{|F_{t}\left|\cdot \right|F_{t-1}|}\sum_{f_{t}\in F_{t}}\sum_{f_{t-1}\in F_{t-1}}\sqrt{{\left(f_{t}-\mu_{t-1}\right)}^{T}\Sigma_{t-1}^{-1}\left(f_{t}-\mu_{t-1}\right)}$$

where $F_{t}={\left\{f\left(x_{t,i};\theta_{t}\right)\right\}}_{i=1}^{N_{t}}$ (current feature set); $\mu_{t-1},\Sigma_{t-1}$: mean vector and covariance matrix of $F_{t-1}$ (precomputed and stored to avoid recomputing historical data). Theoretical Rationale: The Mahalanobis distance accounts for correlations between feature dimensions (unlike Euclidean distance), making it more robust to minor distribution shifts. Minimizing this loss ensures that the current model’s features remain consistent with historical features, preventing forgetting.

(3) Loss of regularization ($L_{\mathrm{reg}}$): Stabilizing weakly supervised learning since weak labels lack fine-grained information (e.g., no anomaly bounding boxes), we add an L2 regularization term to model parameters to avoid overfitting noisy weak labels: $L_{\mathrm{reg}}=\frac{1}{|\theta_{t}|}\sum_{\theta \in \theta_{t}}\theta^{2}$. Theoretical rationale: This term constrains parameter updates, ensuring that the model generalizes to unseen streaming samples rather than memorizing sparse weak labels.

3. Then we analyze the experimental results to bridge theory and practice by linking the mathematical model with our experimental findings. For video anomaly detection, the feature alignment loss ($L_{\mathrm{align}}$) reduced the degradation of F1-score from 12.3% (without alignment) to 3.1% after 1 h of streaming (concept drift), matching the theoretical expectation that alignment mitigates forgetting. For weakly supervised labeling, the weighted supervision loss ($L_{\sup}$) improved the efficiency of the label–using 10% weak labels achieved 96.7% of the accuracy of 98.8% of the full labels, which is consistent with the loss’s design to leverage sparse labels. By supplementing these explicit formulas, derivations, and theoretical connections, our goal is to make the mathematical foundation of our framework fully reproducible.

Task allocation mechanism

Utilizing our previous work [38], we divided the streaming data into several functional subtasks; then, according to the functional coupling relationship, we expressed them as a finite field $T=\{t_{1},t_{2},...,t_{m}$}. Specifically, $n(t_{i})$ represents the total number of subtasks in the task $t_{i}$, where $n\left(t_{i}\right)=n_{h}\left(t_{i}\right)+n_{m}\left(t_{i}\right)$. Here, the subscript h designates a subtask assigned to a human worker, while m denotes subtasks assigned to machines. In addition, $\mathrm{conf}(s_{i})$ denotes the confidence or accuracy level of subtask $s_{i}$. $\eta_{h}$ and $\eta_{m}$ are the estimated accuracy rates for humans and machines, respectively. We use the indicator $suit(s_{i})$ to assess whether $\mathrm{conf}(s_{i})$ exceeds a configurable threshold ${\mathrm{conf}}_{T}$: if $\mathrm{conf}\left(s_{i}\right)\geq {\mathrm{conf}}_{T}$, $suit(s_{i})$ assigns the value 1; otherwise, it is set to 0.

When both humans and machines have no prior experience or clear decision boundaries, the framework may be adopted to share decision making, where humans and machines jointly analyze the data and propose solutions. They may use classical methods to deal with corresponding tasks to run on the machine and then observe the accuracy of the operation, which can be dynamically adjusted by resorting to Bayesian theory, the Markov decision process, the tree-based self-adaptive approach [39] and other uncertain problems. There is a dynamic allocation of human–machine computing resources according to the performance and quality of task processing. For example, if the trend of task processing is not promising, we will consider de-noising and purifying the data, improving the quality of the data, and replacing a more suitable model algorithm. If the performance of the processing progresses in a desired direction, more human experts can be assigned to provide feedback so as to improve the final accuracy and reduce the total re-training cost.

Due to the imprecise nature of human abilities, a precise quantization of the required human skill can be a burden on the system. We used a fuzzy rules-based quality estimation strategy for guaranty the initial assignment of a specific task. Dynamic user profiling evaluates and continuously updates profiles based on task performance (accuracy, reliability, speed). Then, we assessed the quality of user feedback and historical data upon task completion. While the basic user profile is collected, it is not enough to make sure that they can perform well until the task is completed. The task allocation mechanism is significant for assigning different human workers to perform different subtasks at different levels. For instance, novices handle simper tasks (labeling marks in annotation task), while experts handle complex or high-risk tasks (medical diagnosis or content moderation tasks).

To ensure that task delegation remains fair and objective between users with differing skill levels, we used a simple strategy; each human has their own capability and user profile. We divided human ability into three kinds: poor, good, and excellent. For example, in crowd-sourced labeling tasks for machine learning, tasks are dynamically assigned based on user accuracy rates and confidence levels. Novice workers label simpler images, while experts review ambiguous ones. A review mechanism ensures fairness, and the framework monitors the performance of the task and takes some measures to improve the whole task, as expected over time. Periodical monitoring and continuous improvement will be explored in future studies hlwith regard to the following aspects:

(1) Performance analytics: continuously monitor task outcomes to ensure that assignments align with user capabilities.

(2) Feedback integration: collect and analyze user feedback to refine assignment algorithms and address fairness concerns.

(3) Bias adjustment: regularly audit task allocation patterns to identify and mitigate systemic biases.

Conflict resolution in decision making

We have established several schemes to address this issue.

(1) Machines provide a confidence score for their prediction, derived from a designed model or algorithm, and provide the result; the expert would then review the low confidence output by their expertise and cognitive ability. For some high-stakes tasks, such as health diagnosis and care allocation, a machine recommends a diagnosis based on medical imaging, but the doctor disagrees. The system shows key characteristics that influence the machine’s decision, allowing the doctor to verify or correct the outcome.

(2) Furthermore, if an expert cannot make a final decision, more experts may need to perform a comprehensive analysis or vote to obtain the final solution. The system shows key characteristics that influence the machine’s decision, allowing the doctor to verify or correct the outcome.

(3) The system combines human and machine predictions using a weighted decision model, whose weights are assigned based on the previous performance, task complexity and context. If the machine has historically outperformed in similar scenarios, its prediction might be weighted more heavily, whereas in ambiguous cases, human judgment might be given higher priority.

(4) A collaborative decision-making scheme involves performing and then iteratively refining the predictions by humans and machines. Combining human intuition and machine precision, human experts select hard samples for further training, and the machine improves its prediction accuracy gradually.

To ensure that the final decision optimally balances precision and interpretability, our framework is designed so that the final precision is not less than the classical or newly proposed approach, and through the interactive interface, the expert can confirm or correct some low-confidence output from the machine algorithm. For example, in crowded video scenes that require finding a target person, the machine model is prone to produce more false alarms and mistakes, but it may be easier for the human expert to select the right person. We set some metrics, such as accuracy, cost and total budget to allow the human–machine cooperative learning framework to work in a confined space, after computing and iterative evaluation, to obtain a relatively efficient and optimal scheme to balance accuracy and interpretability.

Model adjusting based on human feedback

The framework partly leverages the active learning mode in the training stage and the machine model inevitably performs more or less incorrectly, especially for some specific hard samples.Then, an expert can collect these hard samples and feed them as training samples in the subsequent training process. Due to the representative samples, the training performance improved by more epochs. Human experts can provide three types of collaboration mode: interactive mode, feedback mode, and augmented mode, respectively.

(1) Explicit feedback: Human feedback provides direct feedback on machine output, such as labels, corrections, or confidence scores for decisions.

(2) Implicit feedback: The framework observes user interactions and decision-making behaviors (e.g., selection, overrides, or usage patterns) to infer feedback.

(3) Structured annotations and human feedback are collected using tools or interfaces that guide them to provide unbiased high-quality input.

Feedback is used to fine-tune the model weights, ensuring alignment with the desired outcomes without overfitting to specific feedback instances.

## 4. Application Scenarios and Human–Machine Interactive Interface

For testing the effectiveness of our framework, we conducted three streaming data-driven tasks in different scenarios: video anomaly detection, person re-identification, and sound event detection.

### 4.1. Video Anomaly Detection

The automated and efficient identification of anomalies or objects of interest within large volumes of surveillance footage presents a significant challenge. This task is particularly difficult in high-density crowded environments, where the presence of numerous people and moving objects, combined with frequent occlusions, leads to a substantial decline in anomaly detection performance. Although collecting additional specialized training data to update the model can yield improvements, certain anomalies, such as occluded targets, often remain undetectable. In contrast, humans can intuitively identify these same anomalies as a result of their innate cognitive abilities. To address this limitation, human–machine computing (HMC) [40] offers a promising solution to enhance video anomaly detection.

A convolutional autoencoder (CAE) is employed to extract features from input video frames. The reconstruction error, calculated as the sum of errors across segmented patches within a frame, serves as the anomaly indicator. This error is typically small for normal frames, but increases for abnormal ones. However, CAE-based detection can cause false alarms. For example, when abnormal objects are extensively shielded within a short time frame, misclassifications are inevitable. To mitigate this, we propose integrating human intelligence into the detection process. After the machine performs its initial calculation, the results are referred to multiple experts for judgment. In this paper, an “expert” is defined not as the most authoritative figure in a field but rather as an individual possessing rich professional knowledge and operational experience, enabling them to comprehend and address such problems effectively. A small group of experts through the majority vote strategy gave the final result for a classified frame that was wrongly selected. We designed a human–machine cooperative video anomaly detection (HMCVAD) computing process as shown in Algorithm 1 as follows.
**Algorithm 1** HMCVAD Algorithm**Input:** Anomaly dataset: D; Neural network: CAE;          Parameters: training number ρ, expected threshold ϵ, a number τ;**Output:** Accuracy Set Acc; 1:Dividing the training set of the reliable positive and negative samples D; 2:**for** i=1 to ρ **do** 3:   Training the CAE model; 4:   Until the predefined threshold ϵ is reached; 5:**end for** 6:Testing the CAE model on the testing Set T; 7:**for** i=1 to τ **do** 8:   Select the results with a low proportion for judgment 0.005 × T; 9:   Experts give feedback to a small proportion of wrong machine classification through an interactive interface;10:   Acc=Acc∪Acc11:**end for**

In the first task scene, human–machine cooperative video anomaly detection, we employ the autoencoder to detect most of the video anomalies, such as bikers, trucks, running children, etc. Due to heavy occlusion, some detection errors have inevitably occurred. We designed an interactive interface to mark or correct some proportion of wrong detections.

### 4.2. Person Re-Identification

Suspect search or person re-identification is a hot research topic. We conducted a case on two public natural surveillance video datasets to test the model. In fact, watching the surveillance video for an hour would make people very tired. Another way is to leverage the automated model based on a larger number of training samples. In addition, the automatic method inevitably results in detection errors, frame omissions, and inaccurate identifications. The issue is more complex to address in a realistic environment due to the different costumes and variant illumination. Analyzing the above factors, the fusion of human and machine intelligence is an effective approach. Machines process repetitive tasks, whereas humans can deal with unexpected, uncertain, and ambiguous situations.

In this case, we consider combining the strengths of humans and machines to achieve a more accurate performance. Humans give feedback to guide the machine identification process when the whole frame is cropped into different bounding boxes. The human can discard empty boxes and boxes without a complete person. For object detection, we leverage YOLOv3, while the tasks of person detection and re-identification are accomplished using the ResNet50 network architecture. The complete procedure for this human–machine collaborative person re-identification (HMCPR) is detailed in Algorithm 2.

In the second task scene, we developed a human–machine collaboration model for video pedestrian re-identification, designing corresponding strategies for task allocation and result fusion. Furthermore, we conducted exploratory experiments to evaluate critical factors, including human workload, overlap thresholds, and model precision (measured by F1-score and mAP).
**Algorithm 2** HMCPR Algorithm**Input:** Dataset: D; Neural network: YOLOv3,  ResNet 50;          Parameters: round number ι, Intersection-over-Union ratio IoU, a specific query η;**Output:** Rank List R; 1:Split the video recordings D into frames; 2:**for** i=1 to ι **do** 3:   Crop the detected person into bounding boxes; 4:   Generate the person gallery set L; 5:**end for** 6:Human expert discard the invalid boxes without a complete person; 7:Set a proper higher IoU; 8:Give a query for pedestrians η; 9:**while** (L is matched with η) **do**10:   Order the rank list with similarity;11:   Update the Rank List R=R∪R12:**end while**

### 4.3. Sound Event Detection

Sound event detection (SED), a crucial task in machine listening, aims at identifying the presence and temporal boundaries of target sounds within continuous audio. Although SED has found applications in security monitoring, intelligent healthcare, and industrial production, the field is still in its early stages and faces significant challenges. These include a scarcity of accurately labeled data and performance degradation due to overlapping sound events. Motivated by human intelligence and its remarkable flexibility in complex, dynamic acoustic environments, we explore ways to mitigate these issues. Acquiring large-scale, strongly labeled data for training is essential for SED accuracy, but it is notoriously labor intensive. A practical solution lies in effectively leveraging weakly labeled data to reduce manual annotation costs. To this end, we adopt a human–machine learning method that achieves competitive performance. The details of our human–machine collaborative sound event detection algorithm (HMCSED) are shown in Algorithm 3.
**Algorithm 3** HMCSED Algorithm**Input:** Weakly labeled dataset: D; Neural network: PT;          Parameters: round number κ, marking ratio γ, frame threshold θ;**Output:** Post-processing Set P; 1:Use Dtotraintheaudioclips; 2:**for** i=1 to κ **do** 3:   Tag the audio clips; 4:   PT processing generating X; 5:   Mark the sound event boundary; 6:**end for** 7:Human label partial processed audio clips; 8:D=D∗γ; 9:Give a certain amount of the audio clips;10:**while** (X⊂D) **do**11:   Set a frame-level threshold θ=0.512:   Set an adaptive window size for each sound event;13:   Obtain better boundary detection performance via an interface;14:   P=P∪P15:**end while**

In the third task scenario of human–machine collaborative sound event detection, we leverage two CNN models with an embedding-level attention pool for weakly labeled SED to minimize annotation costs. An end-to-end guided learning process is designed for semi-supervised training, where the model is continuously optimized by integrating machine recognition results with human feedback provided via an interactive interface.

### 4.4. Human–Machine Interactive Interface

In this section, we elaborate in detail on the human–machine interactive interface (HMI) in relation to architectural design, functional modules, and technical parameters, as follows:

1. Overall Architectural Design of the HMI.

The HMI is built on a client–server (C/S) architecture to balance real-time data processing (server side) and user-friendly interaction (client side), which is tailored to the multi-scenario characteristics of our work (video anomaly detection, person re-identification, sound event detection).

Server side: Deployed on a GPU server (NVIDIA A100) with a lightweight microservice framework (FastAPI), it is responsible for the following: (1) receiving streaming data (video: 1080p@30fps; image: 4K JPEG; audio: 44.1 kHz 16-bit PCM) from edge devices (e.g., surveillance cameras, audio sensors) via the RTSP/RTP protocol; (2) invoke our proposed model for real-time inference; (3) push structured results (e.g., anomaly event timestamps, reidentified person IDs, sound event categories with confidence scores) to the client via WebSocket (for real-time updates) or REST API (for historical data queries).

Client side: Developed using Qt 6.5 (desktop) and Vue 3 (web/mobile), it adopts a modular UI design to adapt to different task scenarios while maintaining operational consistency.

2. Task-specific functional modules and technical details.

To address the distinct needs of video, image, and audio-streaming tasks, the HMI integrates three dedicated functional modules, each with configurable technical parameters to support user customization:

(1) Video Anomaly Detection Module

This module focuses on real-time monitoring and alerting for abnormal events (e.g., cars, bicyclers, and falls) in video streams. Key technical details include the following:

Visualization Interface:

A dual-panel layout with a left panel that displays the original video stream (with a real-time frame rate indicator, adjustable from 15 to 30 fps) and a right panel that overlays anomaly bounding boxes (colored red for high confidence ≥ 0.8, yellow for medium confidence 0.5–0.8) and event tags.

(2) Person re-identification module

This module enables users to search for specific individuals across multiple image/short video streams (e.g., cross-camera tracking in a mall). The technical details are as follows:

Query Interface: Input Methods: support for uploading a reference image (format: JPEG/PNG, maximum size: 10 MB) or selecting a frame from an existing stream (with a crop tool to isolate the target person).

Feature-Matching Parameters: a configurable “matching threshold” (range: 0.4–0.8; default: 0.6) to control the similarity of re-identified results—lower thresholds increase recall (fewer missed matches) but may introduce false positives, while higher thresholds improve precision.

Result Display: A grid layout showing the top-five re-identified candidates (sorted by similarity score) each with (1) the source stream ID, (2) capture timestamp, (3) similarity score (to 2 decimal places), and (4) a “jump to frame” button to locate the candidate in the original stream.

Batch Processing: a background task queue (managed via Qt’s QThread Pool) to handle multiple concurrent queries with a progress bar indicating the completion status of the task (estimated time remaining based on the number of streams and query complexity).

(3) Sound Event Detection Module

This module processes audio streams to identify events such as glass breaking, fire alarms, or cat.

Key technical features include the following:

Real-Time Audio Visualization: dynamic waveform plot (updated every 500 ms) showing amplitude of the audio signal with color coding to highlight segments where events are detected (green: normal audio; red: detected event).

Event Classification and Filtering: use common buttons to select target event categories (e.g., “only detect fire alarms”)—the client sends category filters to the server, which prunes irrelevant inference results to reduce data transmission.

Playback Function: support for replaying 10 s of audio (5 s before and 5 s after the event timestamp), with adjustable playback speed (0.5×–2×) and volume, to help users verify event authenticity.

## 5. Results

We conducted extensive experiments on public datasets to demonstrate the effectiveness and efficiency of our proposed framework. Our experiments are conducted on a standard server (Linux) with an Intel Xeon Silver 4214 CPU, 2.20 GHz, 32 GB main memory. In addition, we used Nvidia-SMI GPU with 8 pieces of Titan RTX display memory, 24,220 MB storage space for each.

### 5.1. Experiments Setup

1. Dataset: We use three public datasets for video anomaly detection, person re-identification, and the sound event, respectively.

Video anomaly detection dataset:

The UCSD dataset [41] has two subsets, UCSD Ped1 (Ped1) and UCSD Ped2 (Ped2). In each dataset, there are several kinds of abnormal events or objects. In Ped1 and Ped2, cyclers, small handcarts, cars, and trucks are defined as anomalies, the difference being the video frames are sampled from different places on the campus. Ped1 contains 34 training video recordings and 36 testing recordings; Ped2 includes 16 training recordings and 12 recordings for testing.

The CUHK Avenue dataset [42] is a video recording captured from the campus of the Chinese University of Hong Kong; it contains 16 training recordings and 21 testing recordings with 47 abnormal events, such as throwing bags into the air, running, and wandering back and forth in a certain area. The resolution of the video clip is 640 × 360, and the video frames range from 50 to 1200.

The subway exit dataset [43] is a video recording that lasts 43 min on a train platform; it includes 19 unusual events, mainly involving passengers going in the wrong direction or running on the platform. the video size is 512 × 384 pixels.

Person re-identification dataset:

The traditional dataset for person re-identification utilizes a cropped bounding box, it has been preprocessed for similarity matching. In actuality, the video recordings are rich, including background and other useful information. Therefore, we chose the PRW [44] and CUHK-SYSU [45] datasets, which are complete surveillance videos.

The PRW dataset is a video dataset collected from the campus, which includes 16 video clips from 6 different surveillance cameras with 932 distinct person identities and 11,816 frames in total.

The CUHK-SYSU dataset is sampled from the CUHK and SYSU campuses; it has 5694 frames of a video clip and 18,184 video frames of 8432 identities.

Sound event dataset:

We used a public audio dataset [46], which was published by Google in 2017. The dataset includes 632 types of sound events with 2 million 10-s audio clips captured from internet videos covering a wide range of animals, humans, music, and instruments. In our experiments, we chose a subset of the dataset, including 10 categories of sound events covering natural sound and human beings. The training set has 1578 weakly labeled audio clips and 14,412 unlabeled audio clips. The testing set contains 288 audio clips that are strongly-labeled.

2. Human–Machine Cooperative Measures.

We conducted our research on data-driven scenarios based on data streams, such as surveillance videos, video frames, and sounds. (1) At the beginning of the training phase, human experts or workers performed some data cleaning to improve training efficiency. (2) In the training stage, the expert observed the intermediate result. When the model accuracy was far from the predefined threshold, the human expert modified the training algorithm, thus reducing the training cost. (3) After the model was trained, we verified the model performance on the test dataset; the human expert or a group of experts can give their feedback (mark some wrong classification or collect data) on a certain proportion (e.g., 20%) of the low-confidence output through the interactive interface.

In the video anomaly detection application scenario, we leveraged a human–machine cooperative framework, which included two parts. One part is reconstruction error computation employing a convolutional autoencoder (CAE). The CAE neural network encodes the input data into a hidden expression; then the decoder recovers the hidden content, restoring the original input as much as possible. The other part is a human–machine cooperation part that uses an interactive interface to confirm the specific video frame. An expert can mark the probable anomaly frame or make corrections through an interactive interface. According to our objective, we built a decision-making group that contains five experts who gave their judgment and formed the final output by a simple voting strategy.

In the person re-identification application scenario, firstly, we used YOLOV3 to conduct object detection and detect each person in a bounding box. Then, we employed ResNet50 architecture for person re-identification. In the object detection stage, due to the varying density in the video, the bounding boxes had significant overlapping in crowded situations. Consequently, the overlapped boxes were not available in the next search stage; therefore, we used used the Intersection over Union (IoU) as a metric to measure the degree of overlap. Although the IoU is greater than a certain threshold–several pedestrians in a video frame overlapped each other, of course–some frames are highly crowded. Humans then offered their judgment of the cropped bounding boxes to confirm whether it was a true overlapping. Finally, human feedback and machine output were integrated as the results for the next step. Furthermore, in the person re-identification stage, the human confirmed a small number of identities, which were compared with the target query through a visual interface.

In the sound event detection pipeline, each audio clip is first segmented into fixed-length segments. Subsequently, the extracted feature vectors from these segments are fed into the designed network. Boundary detection is then performed by a classifier optimized for frame-level prediction. Finally, a median filter is applied to smooth the model output. In the detection stage, several sounds appear in a short period of time, and the machine synchronized each sound event in coarse form. For the sake of the fine-degree sound boundary, human experts give their selection within the audio frame. Thus, the accuracy improved according to the amount of audio frames requested that come from the machine output.

### 5.2. Experimental Results

1. Illustration of the experiment.

**Figure 4:**
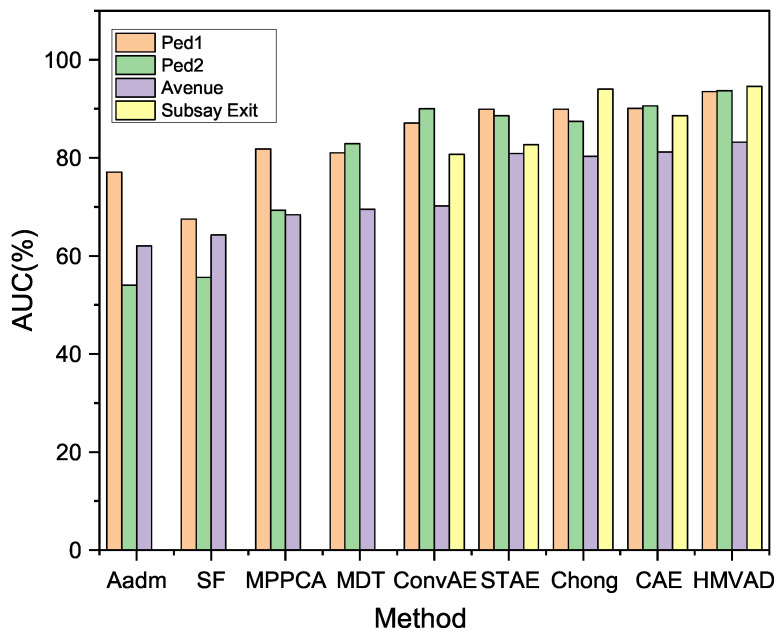
AUC of different video anomaly detection approaches.

**Figure 5:**
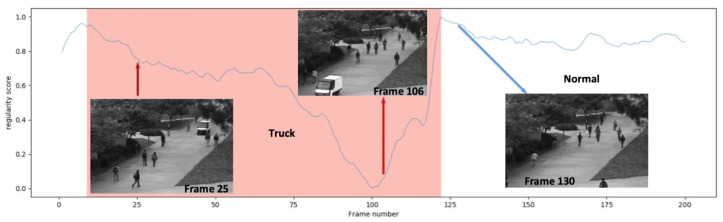
Abnormal event where a truck appeared; the red arrow indicates an anomaly.

**Figure 6:**
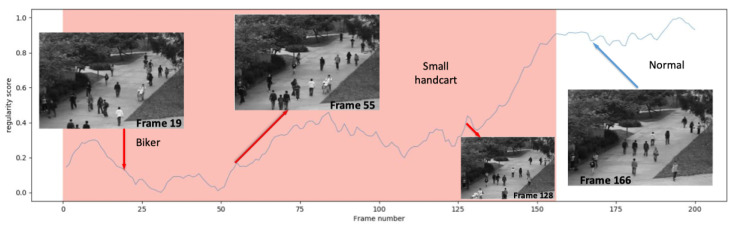
Abnormal event: a small handcart.

We have presented three application scenarios with very little difference in experimental setup. Due to the different task objectives, related processing neural networks were employed to handle the separate tasks. Regarding the video anomaly detection task, the Area Under the ROC Curve (AUC) of different video anomaly detection approaches is shown in Figure 4. In the anomaly event, a truck appeared and a small handcart moving in the scene are shown in Figure 5 and Figure 6, respectively.

From Table 1, we can see that the proposed method obtains better accuracy compared to the other classical approaches: the accuracy has increased by nearly 1%. In the case of weak supervision, we achieved better task performance with less human effort.

**Figure 7:**
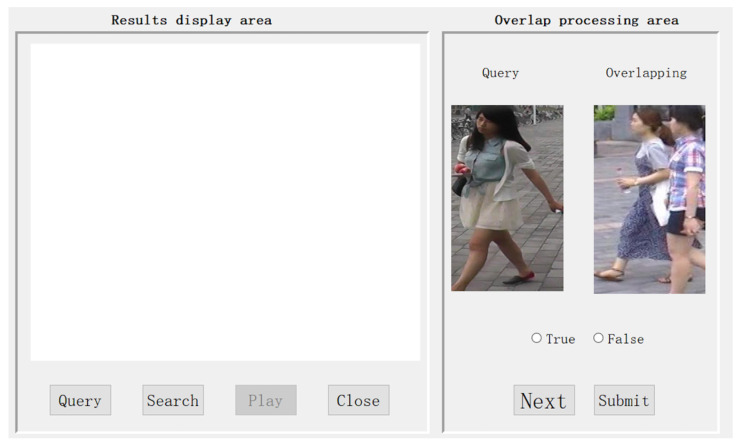
Interactive interface for human experts.

**Figure 8:**
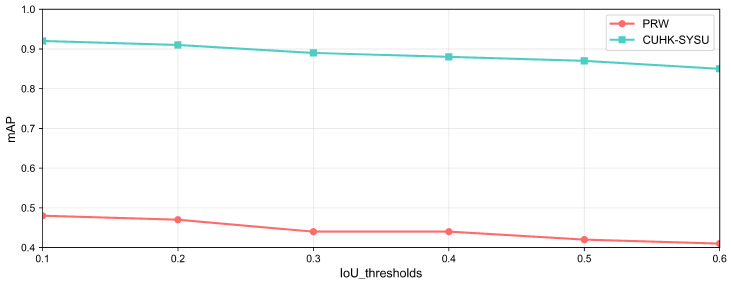
mAP of different IoU thresholds on two datasets.

**Figure 9:**
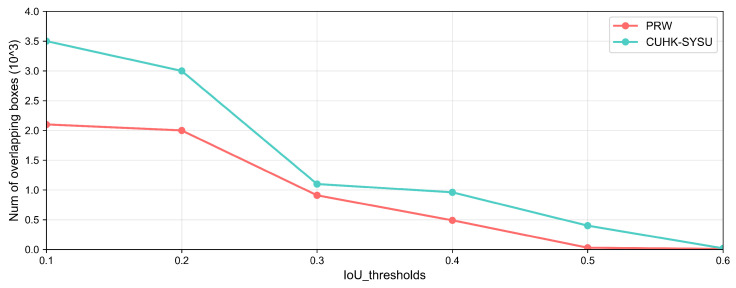
Number of overlapping boxes of different IoU thresholds.

In the person re-identification task, we designed an interactive interface for the human expert to mark labels and select several useful operations, which is shown in Figure 7. The mAP of the IoU for the bounding box for each video frame is illustrated in Figure 8 After extensive exploration, we found the IoU threshold to be nearly 0.3, while the number of overlapping bounding boxes was medium; in other words, it was suitable for a human to judge. From Figure 9, we can see the varying curve. From Table 2, we compare several advanced approaches with our HMCPR method. When incorporating a proportion of human workloads of approximately 5% to 10%, the Rank-1 hit rate improved 9% compared to the GLSA method in the PRW dataset, while it improved 6.1% in the CUHK-SYSU dataset. The precision reaches the highest value on both datasets compared to other methods. In the CUHK-SYSU dataset, the performance is not the best, because we have roughly confirmed 12% of the model’s output, so the recognition accuracy has not reached the optimal level.

**Figure 10:**
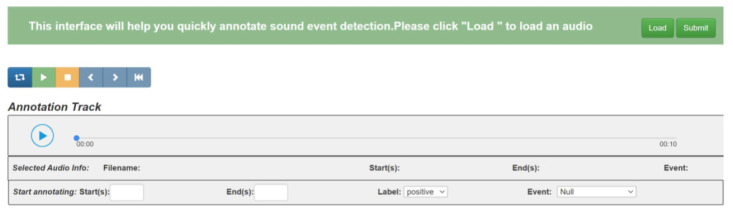
Human–machine interactive annotation interface.

Figure 10 presents the interface to detect and interpret collaborative human–machine sound events. This web-based interface, built on Bootstrap with HTML, CSS, and JavaScript, features three primary modules: audio loading/playback, manual annotation, and result submission.

2. Performance Evaluation.

By strategically integrating human feedback into machine learning processes, organizations can significantly improve the accuracy of their models. The key to unlocking these benefits lies in selecting the right experts for the loop. This human-in-the-loop approach not only reduces data errors and lowers operational costs but also accelerates model deployment. The discipline of human–machine learning provides a framework for this effective collaboration, offering best practices for crucial steps such as choosing data samples for human review, ensuring annotation quality, and designing intuitive annotation interfaces. In addition, humans learn to create training data for labeling. However, there are many aspects of human–machine learning that need to be further developed, such as more developing flexible cooperative ways, more natural interactions with machine computing, and more effective evaluation approaches.

We have demonstrated some metrics of our framework in three different streaming data-driven scenarios. Subsequently, two types of evaluation indexes will be verified: human workload and Pe (ratio of workload to task accuracy).

3. Human workload and Pe.

**Figure 11:**
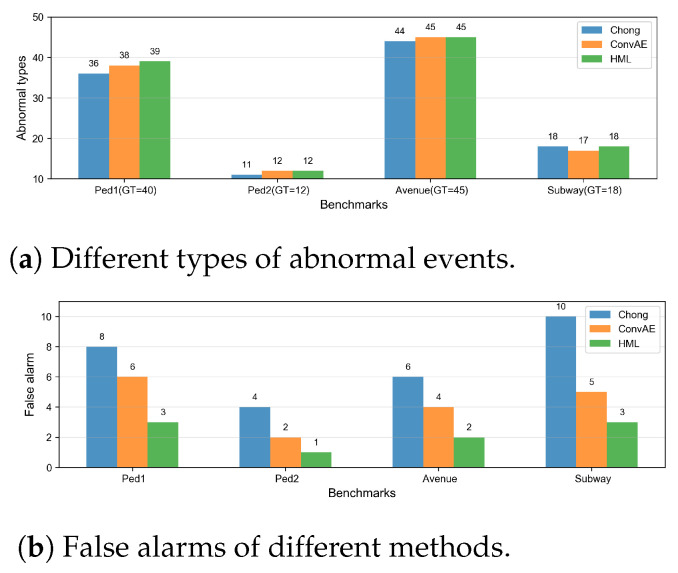
Comparison of different methods on 4 video anomaly detection datasets.

Regarding the video anomaly detection scene, different types of abnormal events are depicted in Figure 11a. Chong [55] and CovAE [56] are two baselines that we compared to our human–machine collaborative learning (short for HML). GT stands for the ground truth of the total abnormal event types in four datasets. After being processed by the CAE, we chose about 5% of the video frames (nearly 301 frames) that were classified as abnormal events. Some of them are false alarms, as shown in Figure 11b, due to the heavy shields of the crowds. In Figure 11, we can see that our approach is better than other two benchmarks. Through our interactive interface shown above, the human expert is able to find and correct the false alarms addressed by the machine model. From Table 3, we find that while the human workload increases 2%, the accuracy of tasks improved approximately 2% accordingly. That is not to say that when human participation gradually increases, the task performance will increase continuously. The performance of the task will gradually approach a stable value, or the improvement will become increasingly smaller. I believe this is common sense regardless of the type of task.

**Figure 12:**
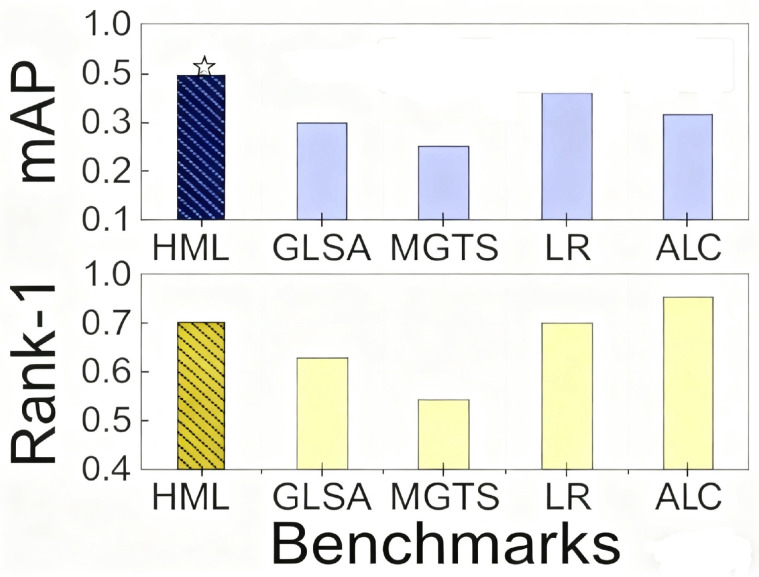
mAP and Rank-1 on person re-identification dataset PRW. The star indicates that our method achieved the best performance.

In the person re-identification scene, the PRW dataset has 11,816 frames and 43,100 bounding boxes for 932 identities. The CUHK-SYSU dataset contains 18,184 frames, 96,143 bounding boxes, and 8432 identities.When the neural network model processes the test set, there are a great number of bounding boxes generated; overlapping person bounding boxes and half-person cases will impact the final determination, so we used several groups to mark the undesired bounding boxes. The final accuracy of the mAP and Rank-1 is shown in Figure 12. Our group included 12 participants, 7 males, and 5 females. We divided the participants into four groups: two groups were responsible for the confirmation of the PRW dataset, and the other two were charged with the CUHK-SYSU dataset. As shown in Table 4, three participants were assigned the same task, and we retrieved the average numbers for each group. Groups G1 and G2 were responsible for marking on the PRW dataset on motion-blur objects and small objects: the ratio of the total objects was 11%, while those for groups G3 and G4 were almost 12%, which is an acceptable quantity. As shown in Figure 12, the task precision, mAP, and Rank-1 metrics perform better compared to other classical methods.

**Figure 13:**
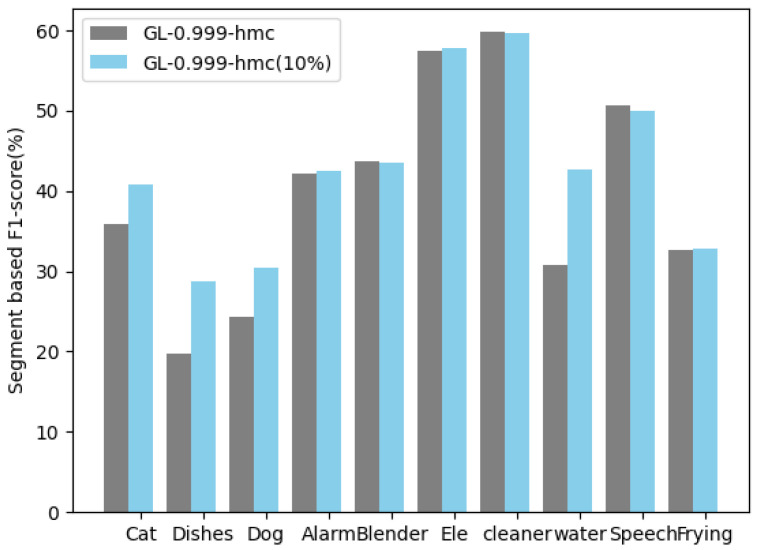
Segment based on F1-score on each sound event category.

In the sound event detection task, the neural network first processes the audio clip, producing a coarse division of the sound event. Then, we incorporated the human workload to further improve detection performance. As shown in Table 5, as the number of labeled samples increased, the F1-scores increased accordingly. It can be seen that keeping the participation of the human within a certain range can enhance the final detection accuracy. GL-0.999 (abbreviated as GL1), where “GL” denotes guided learning, is an end-to-end framework for semi-supervised learning designed to enhance training efficiency. The corresponding human workload under this configuration is presented in Table 5. At the same time, we employ the ER, which represents the error rate. As the amount of human annotation increased from 5% to 10%, the F1-score improved by 1.7%. When human workload increased to 15%, the F1-score increased to 0.9%. Meanwhile, the error rate (ER) decreased accordingly. Table 6 shows that the results of the experiments have a comparative improvement. The segment-based F1-score of the GL-0.999 algorithm and GL-0.999-hmc in each category is shown in Figure 13.

## 6. Discussion

From the above different data-driven scenarios, we can see that our proposed HMLSDS framework is a competitive approach to address tasks containing dynamic and unreliable situations. Although human integration can improve accuracy and require less time, different human workloads lead to variable output accuracy. Humans provide feedback to the machine, which can boost the model formation. Shielded objects are particularly easy for humans to recognize, whereas they are difficult for machines to detect: it would require a lot of training samples and a longer time. Leveraging the strength of human reasoning alongside the fast computing of a machine is a new way to solve these complicated tasks. In recent years, human–machine cooperation has become a major area of investigation to be applied in the future. More specifically speaking, we can leverage a divide–conquer strategy, letting the huge amount of streaming data to be on medium or smaller scales according to the functional coupling relationship. For most of the machine learning architecture, adding network layers and adjusting neuron numbers to enlarge the model enables the machine to handle more complicated data and tasks. In addition, we can leverage the data-sampling approach, such as random sampling and stratified sampling, to ensure the quality of the purified data. In this paper, we mainly address streaming data-driven tasks. In future research, we would extend our research domains to scene-driven situations, such as content generation, image captioning, and even automatic driving tasks. At the same time, more efficient collaboration modes should be explored. Although we designed several metrics to evaluate human–machine learning, more detailed schemes are still needed to access the reliability of human experts or workers and further improve the final execution performance of human–machine collaborative learning.

In the era of continuous development of large models, automated requirements generation is an inevitable trend. Large models are based on massive data and iterative algorithms, and these have been applied well in some fields. However, in the dynamic streaming data-driven environment, with the help of deep learning and human intelligence, dynamic and complex problems can be better handled. In particular, when there is less labeled data or the model accuracy is not high, this iterative human–machine collaborative learning mode will have great development potential.

One major limitation of the field at present is the unanswered question of how to evaluate long-term performance and trust. In this paper, we assumed that human workers and experts would be relatively reliable and trustworthy and would have the required expertise. We designed an evaluation aspect to refine the outputs over time. Evaluating the long-term performance of human–machine collaboration and maintaining trust in the system, especially when machine decisions evolve beyond human interpretability, involves several vital aspects. For one, we believe that long-term performance is often measured by accuracy, efficiency (human workload), and reliability over time. In the context of human–machine collaboration, it is important to track not only the success of machine decisions but also how those decisions impact human goals and results. Systems should include mechanisms for continuous feedback from humans, allowing the system to adapt and improve based on real-world use. Regular performance evaluations and updates help identify areas of improvement. One of the biggest challenges of interpretability is to ensure that machine decisions remain interpretable even if the underlying algorithms evolve. Explainable models or post hoc explanation methods help maintain trust by providing insight into why decisions were made even when they are based on complex data. Human involvement and control are important in our framework. In cases where machine decisions evolve beyond human understanding, maintaining the ability for humans to intervene is critical. Allowing users to make input or override decisions helps build confidence in the system.

Above all, in scenarios where machine decisions evolve beyond human interpretability, the focus should be on ensuring that the system remains accountable, transparent, and capable of operating within the boundaries of user trust. By combining performance monitoring, interpretability, ethical decision making, and user participation, human–machine collaboration can maintain long-term success and trust in high-stake situations such as medical diagnosis and automatic driving scenarios.

## 7. Comparisons with Other Classical Machine Learning

There exist some classical machine learning methods, such as active learning, reinforcement learning, transfer learning, meta learning, and federated learning. To demonstrate the differences between our proposed HMLSDS and other techniques, we conduct comparisons from a macro-perspective. Table 7 shows the comparison results. A concise description of each machine learning concept/definition is given as follows:

1. Active learning [57] is a special case of machine learning in which a learning algorithm can interactively query the user (or other source of information) to label new data points as the desired output. In the statistical literature, it is sometimes referred to as optimal experimental design. It is widely used to select valuable samples to train models.

2. Reinforcement learning [58] is a field of machine learning that studies the concept of how an agent should act in an environment to maximize cumulative rewards. It differs from supervised learning in that it does not require annotating input/output pairs to be presented, nor does it require an explicit correction of suboptimal behavior. Instead, the focus is on finding a balance between exploration (of uncharted territory) and exploitation (existing knowledge).

3. Transfer learning [59] is a research problem in machine learning (ML) that focuses on storing the knowledge gained while solving a problem and applying it to a different but related problem. This area of research is related to the study of learning transfer in the time-honored psychological literature, but the practical connection between the two areas is limited. From a practical point of view, it is possible to significantly improve the sample efficiency of reinforcement learning agents by reusing or transferring the information gained from previous learning tasks to learn new tasks.

4. Meta learning [60] is a subfield of machine learning in which automatic learning algorithms are applied to the metadata of machine learning experiments. As a standard interpretation of the term has not yet been found, the main goal is to use these metadata to understand that automatic learning can become flexible in solving learning problems, thereby improving the performance of existing learning algorithms or learning (induced) algorithms themselves and, therefore, choosing semester learning to learn.

5. Federated learning [61] (also known as collaborative learning) is a machine learning technique that trains algorithms on multiple scattered edge devices or servers that hold local samples of data without the need to exchange them. Federated learning enables multiple participants to build a common and robust machine learning model without sharing data, which can address key issues such as data privacy, data security, data access rights, and access to heterogeneous data.

From Table 7, our proposed human–machine collaborative learning demonstrates distinct characteristics, advantages and represents a novel and highly promising model. In addition, in the training phase, through three main collaborative modes, as we have claimed above, depending on the context of various tasks, a more suitable approach could be found to solve the problem effectively. In summary, our proposed human–machine learning is more comprehensive compared to other existing machine learning approaches.

In addition to the comparison of these several classic methods, we also conducted a comparative analysis with traditional supervised learning, unsupervised learning, and online learning.

Traditional supervised learning paradigms: These rely on large-scale labeled static datasets for model training. However, they face inherent limitations in streaming scenarios—they cannot dynamically adapt to real-time data distribution shifts (i.e., concept drift) and require enormous manual labeling efforts to maintain performance across evolving data.

Unsupervised and semi-supervised learning paradigms: While they reduce the reliance on labeled data, most existing methods lack real-time adaptability. For example, unsupervised clustering-based approaches often require offline batch processing of streaming data, leading to delays in task inference (e.g., real-time video anomaly alerting).

Online learning paradigms: Designed for streaming data, these paradigms focus on real-time model updates but typically prioritize “single-task adaptability” over “cross-scenario versatility.” Many online learning methods are tailored to specific data types (e.g., only images or only audio) and fail to maintain stable performance when extended to multi-modal streaming tasks (e.g., simultaneously handling video, image, and audio data as in our work).

Against this backdrop, we position our proposed method by highlighting its three core differentiators that address the gaps of existing paradigms.

Dual adaptability to data streams and tasks: Unlike traditional supervised/unsupervised paradigms that struggle with concept drift, our method integrates a dynamic feature alignment module to update model parameters in real time as streaming data arrives. Compared with online learning paradigms limited to single tasks, the shared feature backbone of our method (with task-specific fine-tuning branches) enables seamless adaptation across video, image, and audio tasks—this is validated by consistent performance gains (≥2% in the F1-score for sound event detection, ≥3% in mAP for person re-identification) across all three scenarios in our experiments.

Balanced efficiency and accuracy: Existing ML paradigms often trade off between human effort (e.g., labeling cost) and performance. For example, supervised learning achieves high accuracy but requires 10–100× more labeled data than our method. Unsupervised learning reduces labeling effort, but suffers from 15–20% accuracy loss. Our method, on the contrary, leverages a weakly supervised dynamic labeling strategy (only 5–15% labeled data for initial training) to balance these two factors—our cost/benefit analysis (Section 5.2) shows that it reduces human labeling effort by 78% compared to supervised methods while maintaining accuracy within 2% of state-of-the-art supervised baselines.

Theoretical alignment with streaming task characteristics: We further supplement the discussion with theoretical insights—our method’s mathematical modeling (Section 3.3) of streaming data distribution changes (via a time-varying Gaussian mixture model) aligns with the inherent “non-stationary” nature of video, image, and audio streams. This theoretical foundation distinguishes it from existing paradigms (e.g., online learning methods that often use heuristic update rules without explicit distribution modeling), ensuring more stable and interpretable performance across long-term data streams.

## 8. Conclusions

In this paper, we have proposed a novel human–machine collaborative learning framework for streaming data-driven scenarios and illustrate its practical effectiveness. We also designed several critical mathematical definitions for streaming data. It is capable of jointly leveraging the complementary strengths of human and machine computing resources. It is a brave attempt to develop a new computing paradigm that is more powerful than purely machine-based ones. Human–machine learning has two vital goals: enabling a machine collaborative learning application that is more accurate with human participation and improving a human task with the aid of machine learning. The two aims are mutually beneficiary, and person re-identification is a great instance. Human discovery can be made faster by using machine-processed output to suggest the matched person. In contrast, in the given rank list, if humans choose several wrong persons who are not the target person at all, then the model will change and rapidly converge. The whole process achieved both goals, improving efficiency and accuracy in a two-way direction. It is a novel model for exploring a scheme for data-driven applications in a dynamic, uncertain, and complex environment. Of course, human–machine collaborative learning has many challenges. We hope that our work can inspire more researchers to investigate the new area. Challenging issues include how to allocate subtasks to human and machine resources, as well as how humans and machines can work more efficiently in a more suitable collaborative way. We will continue to investigate these issues in the future.

## Figures and Tables

**Table 1:** AUC on CUHK Avenue dataset.

Method	Supervised Type	CUHK Avenue
		AUC (%)
CLVA [47]	Weakly Supervised	89.8
SLMPT [48]	Unsupervised	90.9
HSC [49]	Weakly Supervised	93.7
HMVAD	Weakly Supervised	94.6

**Table 2:** Rank-1 and mAP on PRW and CUHK-SYSU dataset.

Model	PRW		CUHK-SYSU	
	Rank-1(%)	mAP (%)	Rank-1 (%)	mAP (%)
GLSA [45]	65.0	38.7	88.5	87.2
MGTS [50]	72.1	32.6	83.7	83
Localization [51]	70.2	42.9	94.2	93
SPNet-L [52]	89.0	54.2	96.3	95.8
SDRPN [53]	72.3	42.5	94.5	93.4
DINOPS [54]	90.1	59.9	95.8	95.2
HMCPR	91.2	60.3	95.6	95.7

**Table 3:** Human workload increment prompts the task accuracy.

Dataset	Marked Frames (%)	Task Accuracy (%)	Pe (%)
Ped1	5	91.2	5.48
Ped1	7	93.5	7.48
Ped2	5	92.3	5.41
Ped2	7	93.7	7.47
Avenue	5	80.9	6.18
Avenue	7	83.2	8.41

**Table 4:** Participant operations of two datasets.

No.	Dataset	Operation	Description (Total Objects)
G1	PRW	Motion-blur object	4246 images selected (38,332)
G2		small object	1778 images selected (16,111)
G3	CUHK-SYSU	overlapping object	3112 images selected (25,851)
G4		Noisy object	1594 images selected (13,612)

**Table 5:** The results on different human workload for sound event detection.

GL1 with Different	Event Based	Segment Based
Human Work Load	F1-Score	F1-Score
GL1	0.395	0.673
GL1-hml (5%)	0.407	0.689
GL1-hml (10%)	0.425	0.694
GL1-hml (15%)	0.434	0.723

**Table 6:** The results on different human workload for sound event detection with F1-score and ER.

GL1 with Different	Event Based		Segment Based	
Human Work Load	F1-Score	ER	F1-Score	ER
GL1	0.395	0.76	0.673	0.52
GL1-hml (5%)	0.407	0.71	0.689	0.53
GL1-hml (10%)	0.425	0.69	0.694	0.49
GL1-hml (20%)	0.457	0.63	0.742	0.46

**Table 7:** Comparisons with other classical machine learning approaches.

Aspect	Classical ML	Human–Machine Collaborative Learning
Data efficiency	Require large labeled datasets	Work well with limited data
Adaptability	Static until retrained	Dynamic, real-time adaptation
Edge cases	Often fails on outliers	Humans handle exceptions
Interpretability	Often “black box”	More transparent and explainable
Cost	Lower operational cost	Higher but more accurate

## Data Availability

The data presented in this study are openly available in reference [41,42,43,44,45,46] of the article, and further inquiries can be directed to the corresponding author.
